# Superconductivity of boron-doped graphane under high pressure†

**DOI:** 10.1039/c8ra10241d

**Published:** 2019-03-08

**Authors:** Ya Cheng, Xianlong Wang, Jie Zhang, Kaishuai Yang, Caoping Niu, Zhi Zeng

**Affiliations:** Key Laboratory of Materials Physics, Institute of Solid State Physics, Chinese Academy of Sciences Hefei 230031 China xlwang@theory.issp.ac.cn zzeng@theory.issp.ac.cn; University of Science and Technology of China Hefei 230026 China

## Abstract

Based on first-principles calculations, the properties of B-doped graphane under high pressure up to 380 GPa are investigated. We find that B-doped graphane undergoes a phase transition from phase-α to phase-β at 6 GPa. Different from pristine graphane (X. Wen, L. Hand, V. Labet, T. Yang, R. Hoffmann, N. W. Ashcroft, A. R. Oganov and A. O. Lyakhov, Graphane sheets and crystals under pressure, *Proc. Natl. Acad. Sci. U. S. A.*, 2011, **108**, 6833–6837), phase-γ of B-doped graphane is kinetically unstable. The calculated superconducting transition temperature of B-doped graphane at ambient pressure is 45 K, and pressurization can increase the transition temperature notably, *e.g.*, 77 K at 100 GPa. Both the electronic states at the Fermi level and the electron–phonon coupling are mainly contributed by B–C characteristics, indicating that the B-doping plays a key role in the superconductivity.

## Abstract 2

Under high-pressure, the most stable two configurations of B-doped graphane are phase-α and phase-β, and pressurization can increase their *T*_c_ significantly.

## Graphical abstract

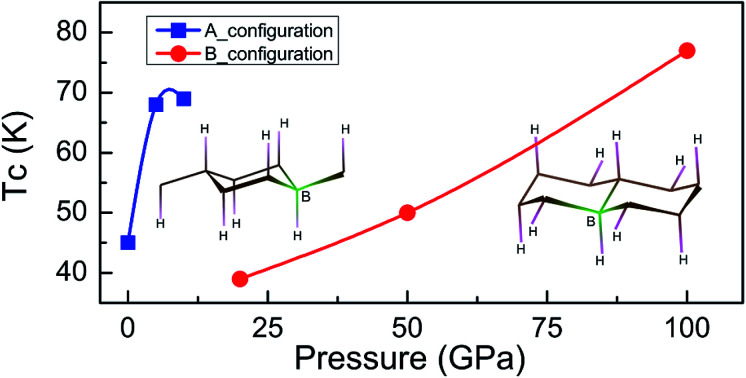

## Introduction

Finding high critical temperature superconductors (*T*_c_) is one of central topics of condensed matter physics and materials science. In the last several years, important progress was achieved in hydrogen-rich compounds. In 2015, under high pressure, H_2_S and H_3_S was identified as high-temperature superconductors with a *T*_c_ of 80 K and 203 K.^1–4^ Recently, *T*_c_ as high as 215–260 K was reported in the La-H_*x*_ system.^5,6^ These hydrogen-rich compounds are conventional superconductors similar to MgB_2_,^7^ in which the superconductivity can be explained by Bardeen–Cooper–Schrieffer (BCS) theory.^8^ In the conventional superconductors, higher vibrational frequency generally gives higher *T*_c_. Since hydrogen is the lightest element, hydrogen in the solid metallic phase will have a high Debye frequency leading to above 3000 K Debye temperature.^9,10^ In addition to hydrogen, carbon in the solid phase can have a Debye temperature of as high as 2230 K,^11^ and it usually forms networks of covalent bonds, such as graphene and diamond. Therefore, high *T*_c_ can also be expected in metallized carbon.

Since boron (B) has one electron less than carbon, B dopant is an effective way to realize the metallization of carbon networks with hole-doping.^12–16^ Boron dopant is also widely used to extend the applications of carbon materials, for example, B doped graphene has important applications in the fields of lithium storage^17,18^ and catalysts^19^ Furthermore, since the electronic states of the B–C sigma bonds will cross the Fermi level. For example, B dopant can turn diamond into superconductor with the *T*_c_ of ∼4 K,^12^ where sigma bond plays an important role.^20^ The p-type doping in organic materials can induce superconductivity, such as diamond-like crystalline hydrocarbon,^21^ hydrogenated carbon nanostructures,^22^ and polyethylene,^23^ and they all have noticeable superconducting transition temperatures. Actually, in the carbon based low-dimensional materials, such as graphene and carbon nanotubes,^15,24,25^ much stronger electron–phonon coupling (EPC) has been proved. Theoretical results show that B-doped graphene will have high *T*_c_ ranging from 49 K to 72 K.^25^ Furthermore, based on the uniform hole-doping model by removing electrons from the system, maximum value of *T*_c_ = 96 K was reported in hole-doped graphane at ambient pressure.^16^

In spite of doping, pressure is another important and effective route to achieve higher *T*_c_ in the conventional superconductors, for example, pressure-induced high *T*_c_ in H_*x*_S systems.^1,2,4,26,27^ This is partially because that pressure can increase the phonon frequencies and induce some new phases. However, the effects of pressure on the superconductivity behaviors of carbon based two-dimensional materials have not been clarified yet. Does the pressure can also increase the *T*_c_ of carbon based two-dimensional materials notably?

In this work, we investigate the superconductivity of B-doped graphane under high pressure. The phase transition of B-doped graphane under high pressure is examined at first. Then, the properties of superconductivity are analyzed, which shows that the *T*_c_ of B-doped graphane can be notably increased from 45 K to 77 K by pressure. The discrepancies between chemical doping (B dopant) and ideal uniform hole-doping (remove electrons from the system)^16^ has been clarified, where localized hole distribution exists in the case of B dopant but even (homogeneous) hole distribution occurs in the ideal uniform hole-doping model.

## Methods

**Fig. 1:**
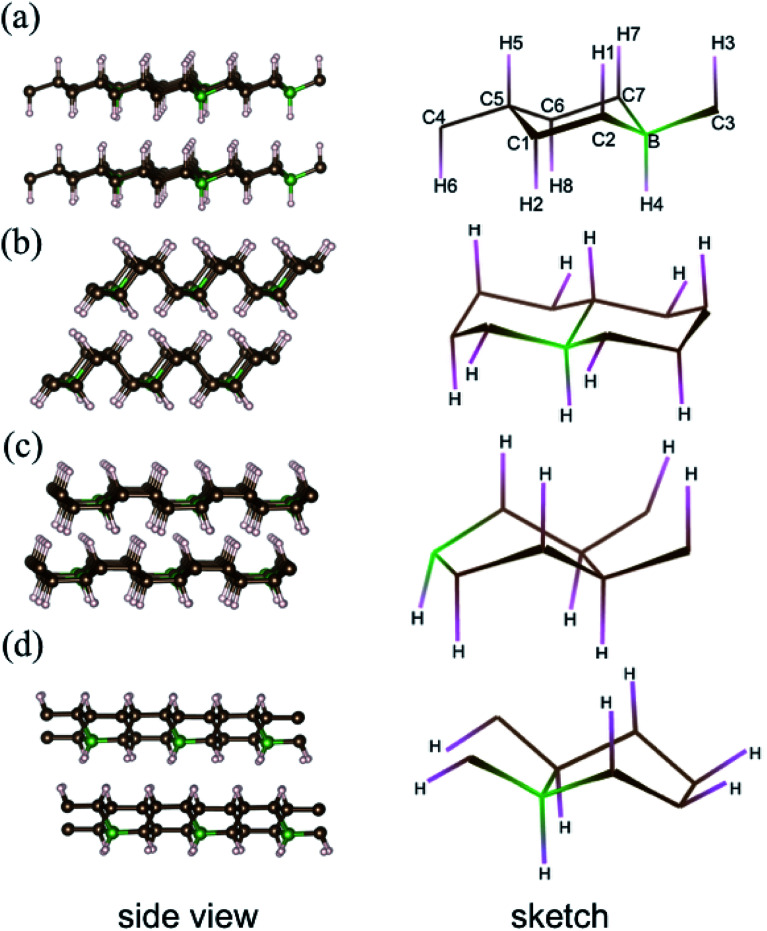
Configurations of B-doped graphane in AA-stacking phase-α (a), -β (b), -γ (c), and -δ (d). Left part: side view. Right part: sketch diagram. C, H, and B atoms are shown in brown, pink, and green, respectively. In the sketch of phase-α, the atom positions are marked with Arabic numerals.

Previous simulations pointed out that for graphane, there are four stable phases with different carbon ring configurations (chair-like or boat-like) and C–H bond orientations.^28^ These graphane phases are more stable than benzene from ambient pressure to high pressure (300 GPa),^29^ and the enthalpies of several CH phases are shown Fig. S1† as a function of pressure. We introduce 12.5 mol% B dopants in these four stable phases, which are shown in the Fig. 1 as phase-α, phase-β, phase-γ, and phase-δ, respectively. Phase-α and phase-β are chair-like configurations. Seeing from the right panel of Fig. 1(a), neighboring C–H bonds locate at opposite side in phase-α. However, in phase-β (Fig. 1(b)), three adjacent C–H bonds orientate to the same direction, and the other three C–H bonds orientate in opposite directions. The other two phases are boat like, orientations of C–H bonds or B–H bonds are displayed in the sketches of each phase in the right part of Fig. 1.

Structural optimization, enthalpies, and electronic structures were calculated using the Vienna ab initio simulation (VASP) code^30–32^ with projector-augmented plane-wave (PAW) potentials. We employed 1s^1^, 2s^2^2p^2^, 2s^2^2p^1^ valence states for H, C, and B potential, respectively. The energy cutoff was set to 800 eV in all calculations, and 2 × 2 × 2 supercells of graphane were used. The *k*-point grid was generated by Monkhorst–Pack scheme and ensured that the energies converged to within 1 meV per atom. The forces were converged to less than 0.001 eV Å^−1^. EPC were calculated using Quantum Espresso (QE) code^33^ with density-functional perturbation theory (DFPT).^34^ The cutoff energies for wave functions and charge densities were set to 40 Ry and 240 Ry, respectively. We used 2 × 2 × 2 *q*-point meshes for EPC parameter λ, and denser *k*-point meshes 8 × 8 × 8 for electron–phonon interaction matrix element calculation.

## Results and discussion

**Fig. 2:**
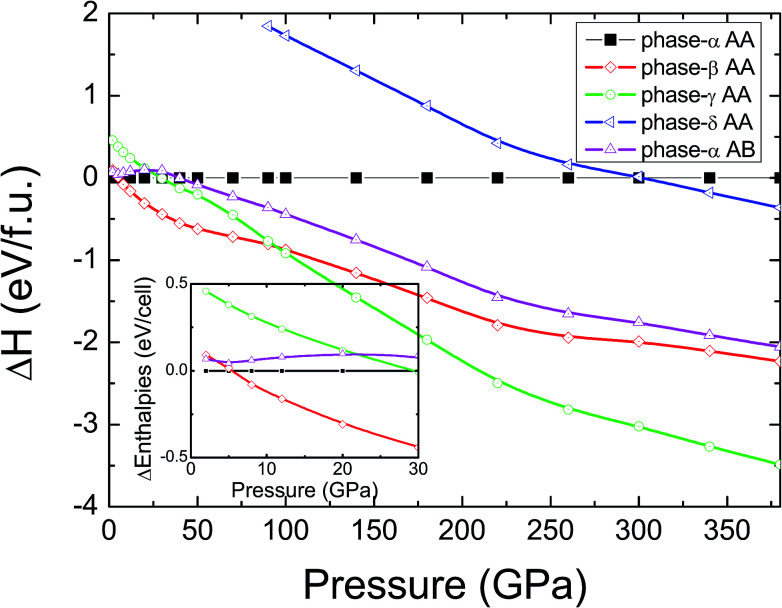
Calculated enthalpies per C_7_BH_8_ cell as function of pressure are shown by taking the enthalpy of phase-α in AA-stacking as reference. AA(AB) indicates the AA(AB)-stacking profile. The inset exhibits the zoom in image of low pressure part (0 GPa < *P* < 30 GPa).

In pristine bulked graphane, AA-stacking features were proved to be more stable than AB-stacking cases.^28^ To check the stability of AA-stacking or AB-stacking in B-doped graphane, we calculated enthalpies of phase-α in AA-stacking and in AB-stacking. The calculated enthalpies are shown in Fig. 2. The inset of Fig. 2 zooms in low pressure range, and it shows that the enthalpy of phase-α in AA-stacking is smaller than that of phase-α in AB-stacking, indicating that similar to pristine graphane, B-doped graphane in AB-stacking is metastable below 40 GPa. Therefore, we will only consider the stability of phase-α, phase-β, phase-γ, and phase-δ in AA-stacking profiles, and the calculated enthalpies of these phases are shown in Fig. 2 as function of pressure.

We can find from Fig. 2 that the most stable phase in the pressure range 0–6 GPa is the phase-α in AA-stacking, which has a symmetry of *P*3*m*1. Above 6 GPa, phase-β becomes more stable than phase-α, and phase-β keeps the stability up to 95 GPa. Upon further compression, phase-γ has the lowest enthalpy at the pressure higher than 95 GPa. Phase-δ is not competitive in enthalpy with other configurations in the whole pressure range from 0 to 380 GPa. Moreover, imaginary frequencies are observed in the phonon dispersions of phase-γ, indicating that it is kinetically unstable. Therefore, for B-doped graphane, phase-α (0–6 GPa) and phase-β (6–380 GPa) are two stable phases under pressure. The phase transition of B-doped graphane is different from that of pristine, which undergoes phase transition from phase-α to phase-β at 15 GPa then to phase-γ at 240 GPa.^28^ For comparison, we calculated the phase transition of 6.125 mol% B dopants in these three phases (see in Fig. S2†), we can find that the phase transition sequence of 6.125 mol% B-doped graphane is constant with the case of 12.5 mol%-doped case. The critical pressure of 6.125 mol% B-doped graphane from phase-α to phase-β is 11 GPa, then transform to phase-γ at 123 GPa. The critical pressure points fall in between pristine and 12.5 mol% B dopants graphane.

**Fig. 3:**
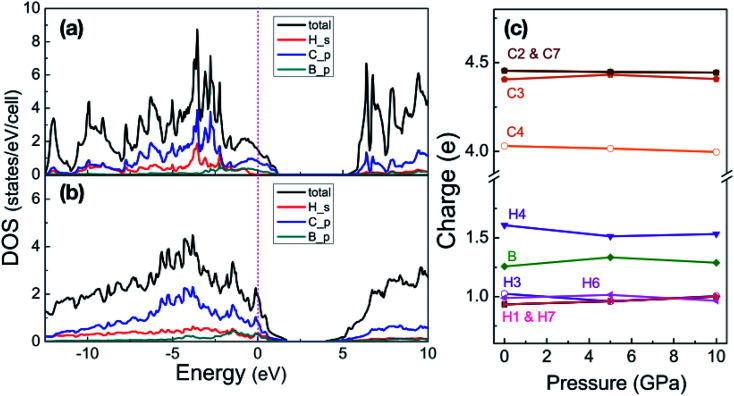
Partial density of states of phase-α at 5 GPa (a) and phase-β at 100 GPa (b). Energy is respected to the Fermi level. (c) Shows the Bader charges of atoms in the phase-α are shown as a function of pressure, the numbers match the sketch of phase-α in Fig. 1.

With pressure increasing, distance between atoms will change, and the results are summarized in Table S1,† will change. The B–H bond length (*d*_B–H_) in B-doped graphane keeps decreasing from 1.208 Å (phase-α at ambient pressure) to 1.150 Å (phase-β at 100 GPa). On the other hand, C–H bond length (*d*_C–H_) is slightly shorter (0.1 Å) than *d*_B–H_ under pressure. From Bader charge analysis of phase-α (see Fig. 3(c)), we can see that the electrons of B atom transform to neighbor atoms (H and C), since the electronegativity of B is weaker than C and H. The length of C–C bond next to B atom is shorter than that of C–C bond far from B atom, because of the charge transformation of B to C. Furthermore, over the whole investigated pressure range, B-doped graphane crystal keeps layer stacking and the layer distance represented as *d*_H–H_ in Table S1† decreases with pressure increasing.

We now inspect the electronic structure of the most stable phases – phase-α and phase-β. The partial density of states (PDOS) of phase-α at 5 GPa and phase-β at 100 GPa shown in Fig. 3(a) and (b) illustrate that B-doped graphane in both phase-α and phase-β are metal. The electrons of C p-orbitals and B p-orbitals make the most contribution to the density of states but not those of H s-orbitals indicating that the metallization origins from the C–B rings. Moreover, the H s-orbitals electrons contribute to the superconductivity of phase-β, since H s-orbitals cross the Fermi level. As shown in Table 1, density of states at the Fermi level increases slightly with pressure increasing in phase-β, *e.g.*, the electronic states increase from 8.50 states per cell at 20 GPa to 10.96 states per cell at 100 GPa.

**Table 1:** Superconducting properties of B-doped graphane under pressure. N_EF_ is the electronic states at the Fermi level

Phases	Pressure (GPa)	*λ*	N_EF_ (states per cell)	*ω*_log_ (K)	*T*_c_ (K)	
					μ* = 0.10	μ* = 0.13
Phase-α	0	1.62	9.66	369	45	41
	5	1.52	9.75	588	68	62
	10	1.26	9.53	730	69	62
Phase-β	20	0.92	8.25	644	39	33
	50	1.00	10.16	718	50	43
	100	1.72	10.96	602	77	71

**Fig. 4:**
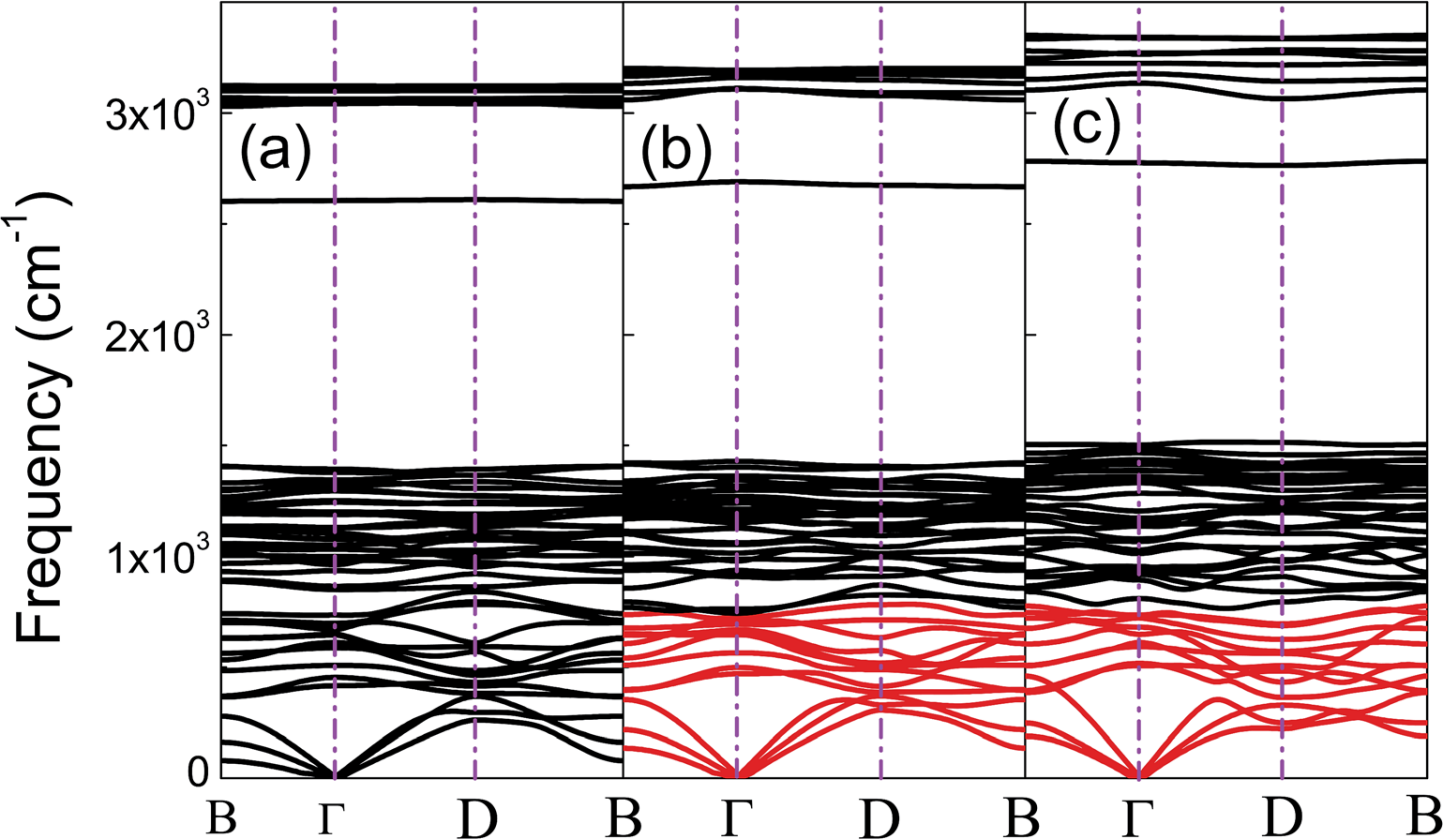
(a), (b), and (c) show the phonon dispersions of phase-β at 20 GPa, 50 GPa, and 100 GPa, respectively. The softened phonon branches at D point are shown in red at (b) and (c).

**Fig. 5:**
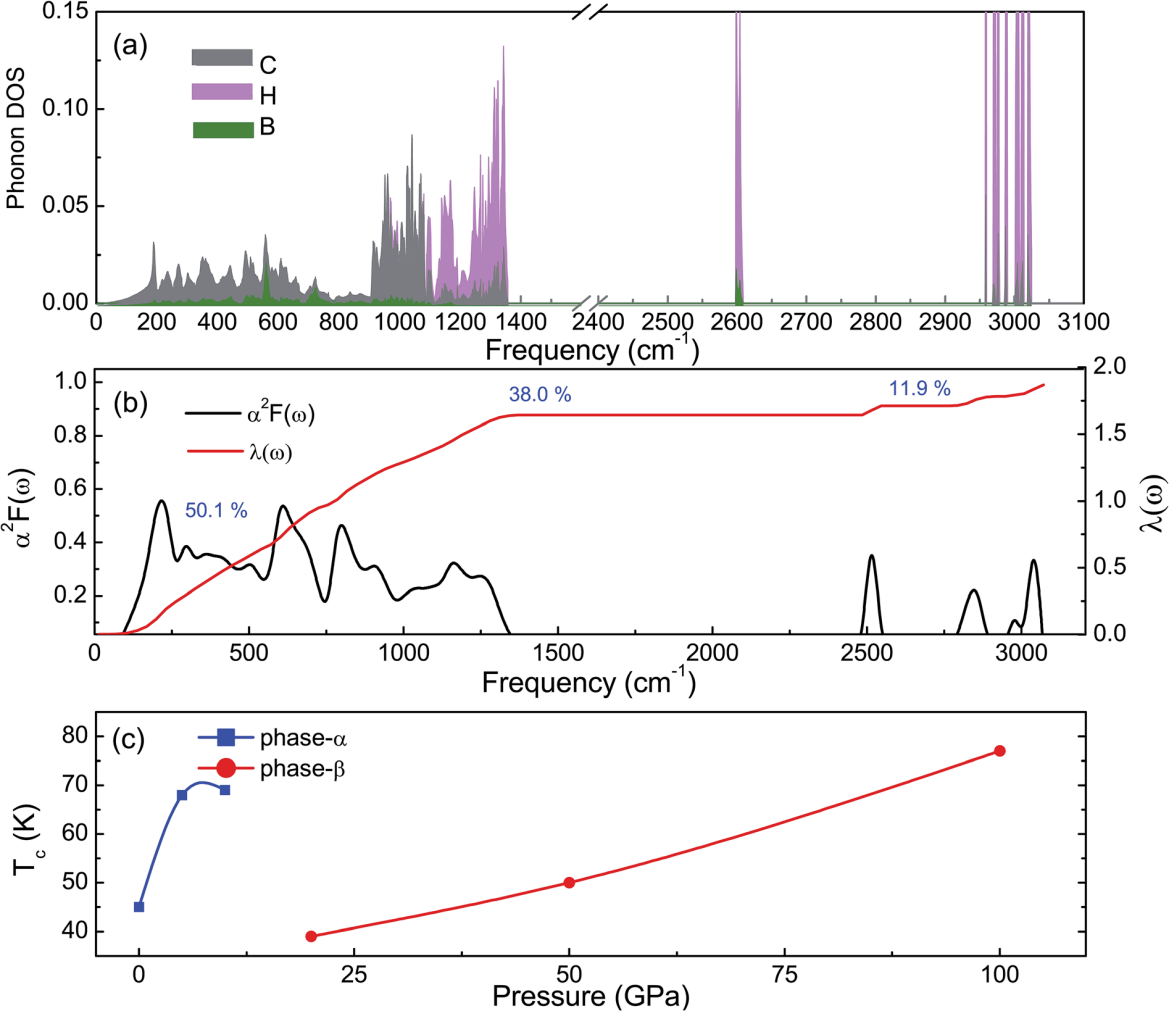
(a) Partial phonon density of states of graphane at 10 GPa. Gray, pink, and green represent C, H, and B atoms, respectively. (b) Shows the Eliashberg phonon spectral function *α*^2^*F*(*ω*) and electron–phonon integral *λ*(*ω*) of phase-β at 100 GPa. (c) Shows the *T*_c_ of B-doped graphane is shown as function of pressure.

The phonon dispersion curves of phase-α (see Fig. S2†) and phase-β (see Fig. 4) under pressure were calculated to explore the lattice dynamics of B-doped graphane. The absence of imaginary frequencies implies the kinetically stability of phase-β under high pressure. The whole phonon density of states of phase- α at 10 GPa can be divided into three parts, which is shown in Fig. 5(a). The low-frequency vibration below 750 cm^−1^ mainly comes from the C–C or C–B stretching vibrations, C–H and B–H bonds shear motions contribute to the middle-frequency vibration (750–1500 cm^−1^).^16^ After a large gap, C–H and B–H bonds stretching mainly contribute to the highest frequency area above 2500 cm^−1^. Among this area, B–H stretching vibration appears around 2600 cm^−1^, while C–H stretching mode sites around 3000 cm^−1^. Phase-α and phase-β at all calculated pressure points have similar profiles in phonon dispersion, all of them can be divided into three parts in frequency.

Taking phase-β at 100 GPa for example, from the Eliashberg phonon spectral function *α*^2^*F*(ω) and integrated EPC parameter (*λ*) as shown in Fig. 5(b), the low-frequency (blow 750 cm^−1^) and (750–1500 cm^−1^) middle-frequency vibration contributes as much as 50.1% and 38.0% of total electron–phonon coupling constant, respectively. The remaining *λ* comes from high-frequency C–H and B–H stretching motions. This result indicates the significant role of low-frequency and middle-frequency vibrational modes. However, C–H and B–H stretching motions contribute less to electron–phonon interaction. Namely, B–C rings play an important role in the superconductivity of B-doped graphane.

Based on the *λ* and logarithm of average frequency (*ω*_log_), we employed the modified McMillan equation by Allen and Dynes to calculate the *T*_c_.1
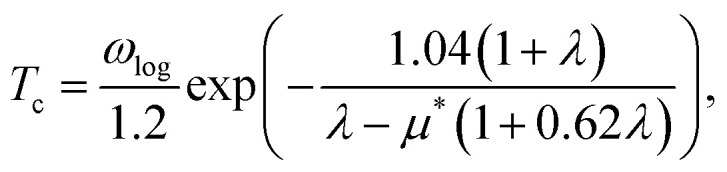
where μ* stands for the screened Coulomb repulsion between paired electrons,^9^ and it is typically chosen between 0.10–0.15. We chose μ* value 0.10 and 0.13 in this work. The calculated parameters related to superconductivity are listed in Table 1, where N_EF_ represents the electronic states at the Fermi level. We can find that the N_EF_ of phase-α at 10 GPa is smaller than that of 5 GPa, but larger than that of phase-β at 20 GPa. Since the electrons of H s-orbital contribute to N_EF_, the N_EF_ of phase-β at 50 GPa and 100 GPa are much larger than that of phase-α under pressure. Take phase-β at 100 GPa for instance, the *λ* is 1.72 and *ω*_log_ is 602 K, leading to that the calculated *T*_c_ using modified McMillan equation is 77 K.

To clarify the pressure effects on the superconductivity of B-doped graphane, we calculated the critical temperature of superconductivity of both phases. The *T*_c_ of B-doped graphane at the pressure ranging from ambient pressure to 100 GPa was calculated, and the results are presented in Fig. 5(c). At ambient pressure, the *T*_c_ of phase-α is 45 K, while it increases to 68 K at the pressure of 5 GPa. The *T*_c_ at 10 GPa (69 K) is comparable with that of 5 GPa, and the reason is that from 5 GPa to 10 GPa, the decrease of *λ* counteracts the increase of *ω*_log_ as shown in Table 1. It is noticeable that the *T*_c_ of phase-α we simulated at ambient pressure is 45 K, much lower than that (96 K) predicted by Savini *et al.*^16^ The reason lead to this large divergence mainly comes from the charge distribution difference between chemical doping and ideal uniform electronic doping. In the ideal uniformed electronic doping model by removing electrons from the investigated system, due to the nesting of the Fermi surface, large medium frequency phonon soften appears at gamma point.^16^ As shown in Fig. S4,† we obtain similar phonon soften in the graphane by removing 12.5% electrons. However, in the case of chemical doping, the charge is localized around B atom but not uniformly distributed in real chemical doping mode of phase-α. It can be found in Fig. 3(c) that charges of B atom transform to atoms nearby (C and H). That is the reason that we did not find the medium frequency phonon soften at gamma point as shown in Fig. 4. In phase-β, the *ω*_log_ of phase-α at 20 GPa and 50 GPa is 644 K and 718 K, respectively. However, it decreases to 602 K at the pressure of 100 GPa. The decrease of *ω*_log_ from 50 GPa to 100 GPa origins from the soften of phonon at the D point (Fig. 4 (b) and (c)) induced by pressure. The pressure-induced phonon softening is a common behavior of superconductors at high pressure,^35–37^ and it plays a crucial role in enhancing the electron–phonon coupling.^1,38,39^ Therefore, the *λ* of phase-β increases with the pressure increasing, which counteracts the decrease of *ω*_log_. We can find that, over the pressure range from 20 GPa to 100 GPa, the *T*_c_ of phase-β almost monotonously increases from 39 K to 77 K. Our results show that the *T*_c_ of each B-doped graphane phase will notably increase with pressure increasing. The B–C bonds make the most contribution to *T*_c_ in both phase-α and phase-β, and upon further compression, H s-orbitals electrons in the phase-β will contribute more to the metallization and superconductivity of B-doped graphane. For the lower doping concentration, *e.g.*, 6.125 mol% B-doped graphane, the electronic states at the Fermi level will become smaller. Therefore, similar to the concentration effect of hole-doped graphane,^16^ lower B concentration may lead to lower superconducting critical temperature.

## Conclusions

We have investigated the phase transition, metallization, and superconductivity of B-doped graphane in the framework of density functional theory. We found that at low pressure (0–6 GPa), B-doped graphane in phase-α with AA-stacking is the most stable phase, and AB-stacking are metastable configuration. Phase-β is the most stable case within the pressure ranging from 6 to 380 GPa. All B-doped graphane phases are metal, and B–C and C–C vibrations dominantly contribute to the electron–phonon coupling. At ambient pressure, because of the Fermi surface nesting induced phonon softening, the ideal uniform hole-doping model by removing electrons from graphane gives higher *T*_c_ than that of the B-doped case. Moreover, pressure-induced phonon softening in B-doped graphane is observed, and pressure can significantly increase the *T*_c_ of B-doped graphane in both phase-α and phase-β.

## Conflicts of interest

There are no conflicts to declare.

## Supplementary Material

RA-009-C8RA10241D-s001
