# Supplementary material for: Superconductivity of boron-doped graphane under high pressure
Source: RSC Adv. 2019 Mar 8;9(14):7680–6. doi: 10.1039/c8ra10241d (PMC9061170; doi:10.1039/c8ra10241d)
Supplement: RA-009-C8RA10241D-s001 [file RA-009-C8RA10241D-s001.pdf]

Electronic Supplementary Material (ESI) for RSC Advances.
This journal is © The Royal Society of Chemistry 2019

Superconductivity of boron-doped graphane under high pressure

Ya Cheng[†,‡], *Xianlong Wan*[*,†,‡], *Jie Zhang*[†], *Kaishuai Yang*[†,‡], *Caoping Niu*[†,‡], *Zhi Zeng*[*,†,‡]

† Key Laboratory of Materials Physics, Institute of Solid State Physics, Chinese Academy of Sciences, Hefei 230031, China

‡ University of Science and Technology of China, Hefei 230026, China

*E-mail: xlwang@theory.issp.ac.cn and zzeng@theory.issp.ac.cn

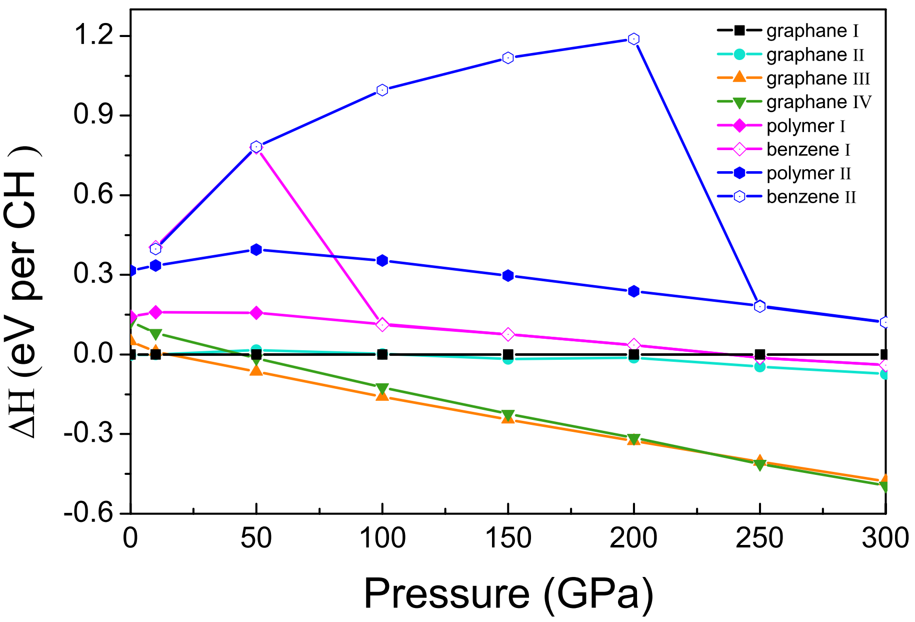

Figure S1. gathered enthalpies per CH as function of pressure are shown by taking the enthalpy of graphane I as reference.

In Figure S1, graphane I to IV are the same phases in PNAS 108:6833-6837 (2011), benzene I and benzene II are the benzene phase III and benzene phase V in JACS 133:9023-9035. This plot indicates that the phases of pristine graphane are more stable than benzene under high pressure.

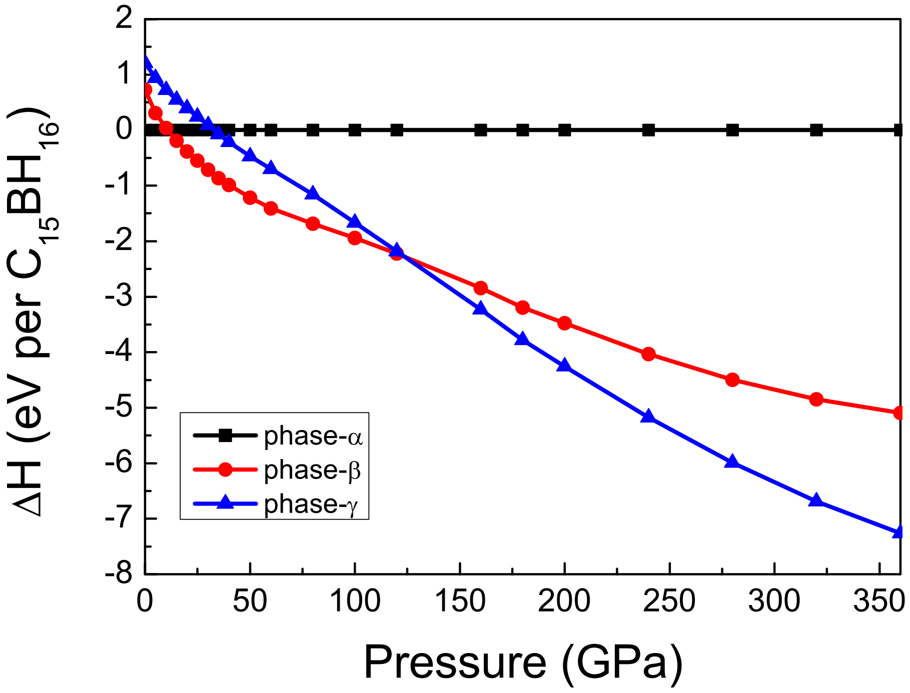

Figure S2. Calculated enthalpies of 6.125 mol% B dopant graphane as a function of pressure in phase-α, phase-β, and phase-γ, and phase-α is taken as a reference.

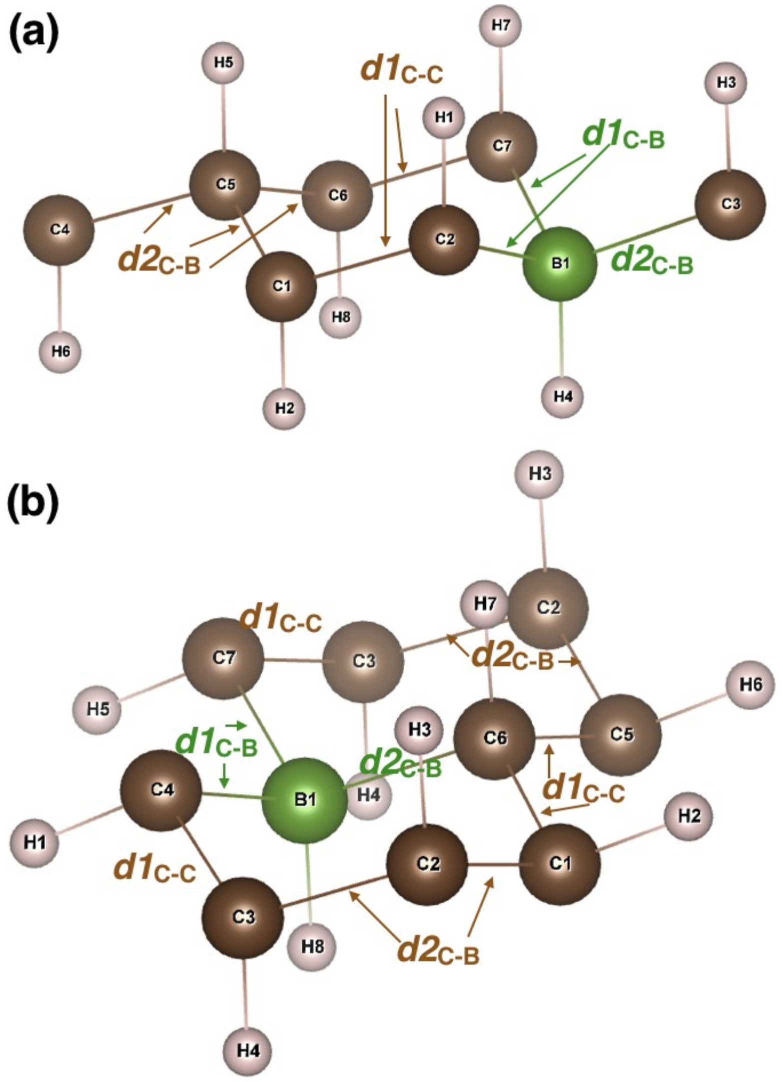

Figure S3. Configurations of phase-α (a) and phase-β (b). Distances between atoms are also named in each figure.

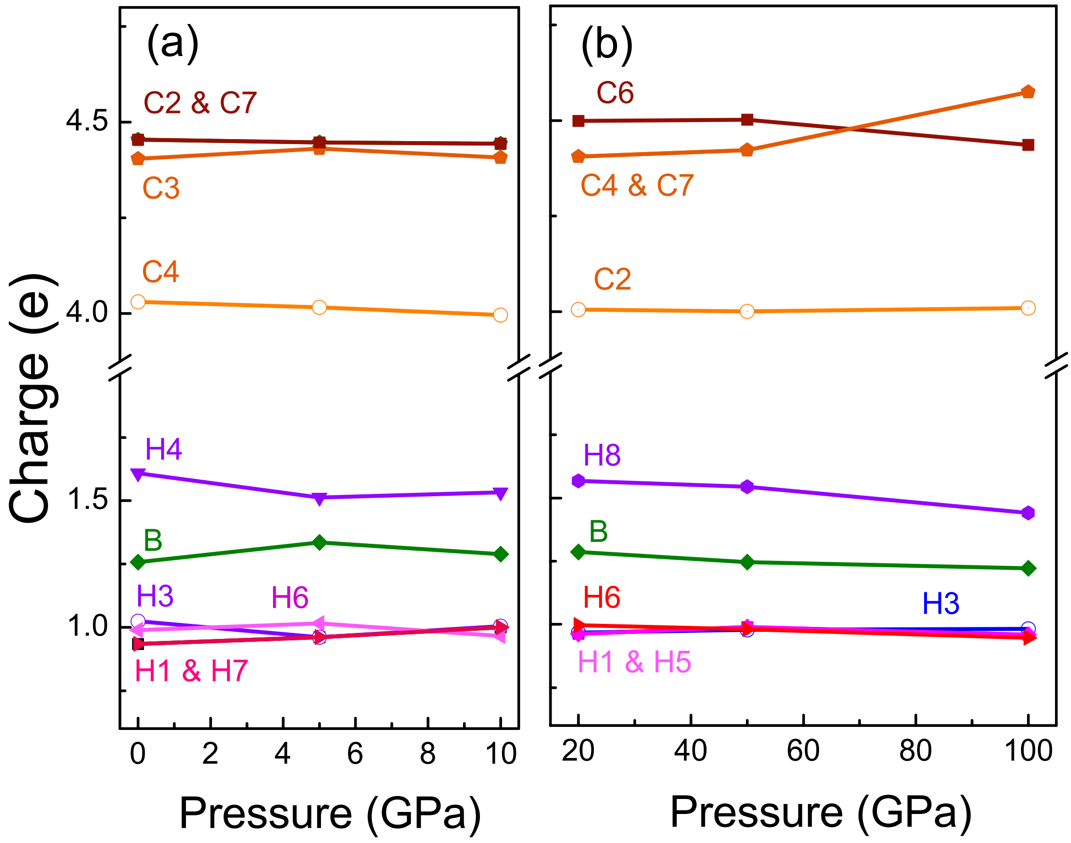

Figure S4. Bader charge of C and H atoms next to B atom as a function of pressure. The structures of phase-α (left) and phase-β (right) are shown as insert graphs. The charges of further C atoms keep to be near 4 e, as orange lines were shown in graph (a) and (b).

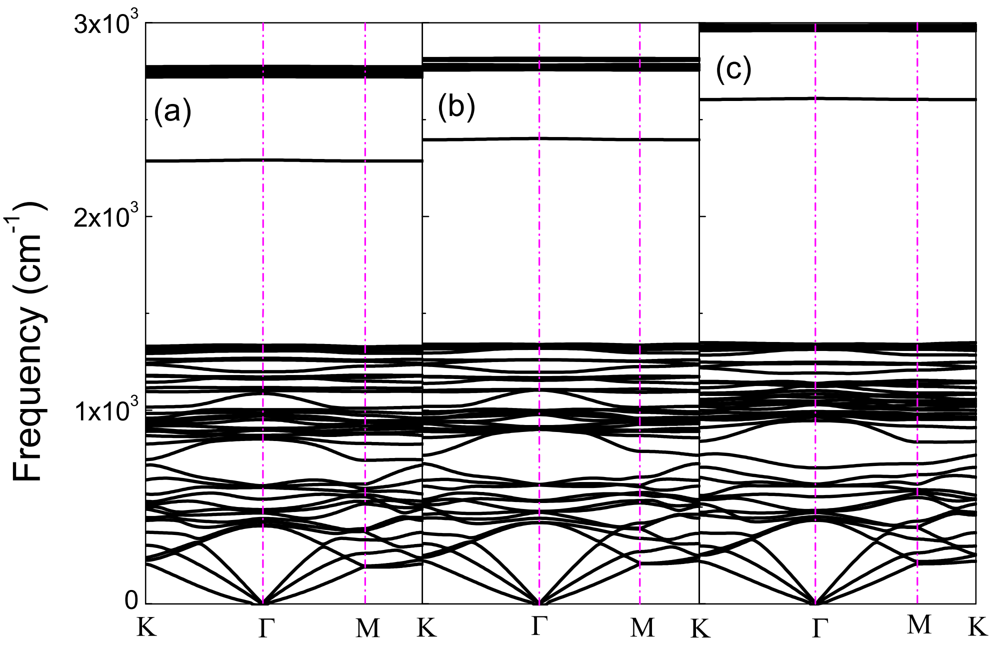

Figure S5. (a), (b), and (c) show the phonon dispersions of phase- α at 5 GPa, 10 GPa, and 200 GPa, respectively.

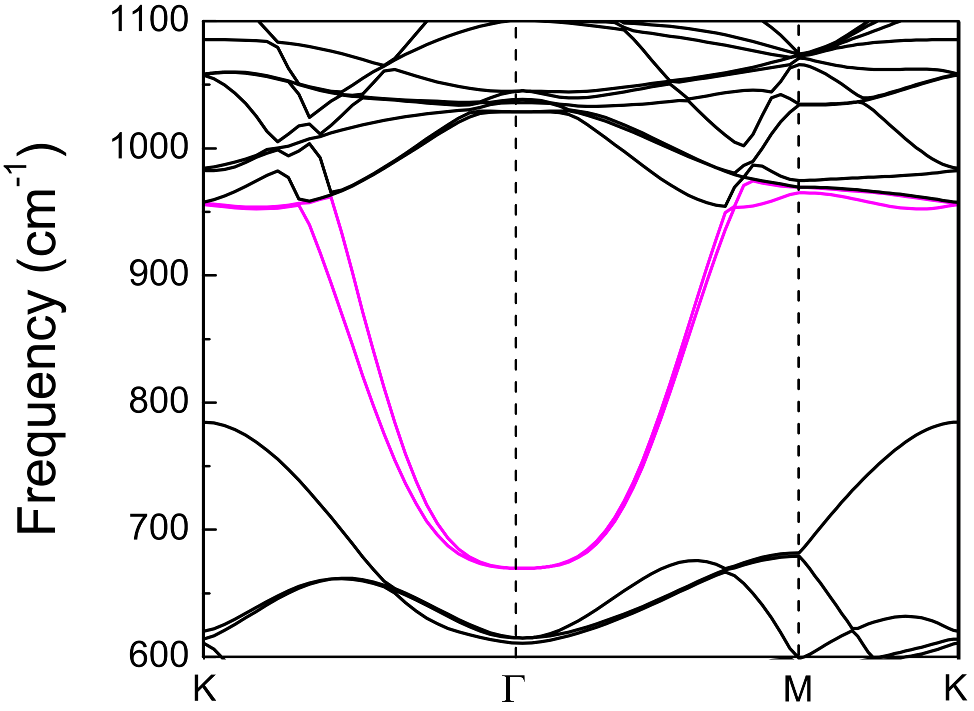

Figure S6. Phonon dispersion of 12.5 % hole-doped phase-α graphane under ambient pressure based on the ideal uniform hole doping model by removing electrons from graphane. The softened phonon branches at Γ point induced by the nesting in fermi surface are shown in pink.

Table S1. Distances changes under several typical pressure points between atoms. (For phase- α, three C-B bonds are equivalent, so $d1_{C\text{-}B}$ and $d2_{C\text{-}B}$ are equal. $d1_{C\text{-}C}$ represents the distance of C1-C2 or C6-C7, $d2_{C\text{-}C}$ is the distance between C5 and its neighboring C atoms. For phase-β there are two equal C-B bond length, another C-B distance between B and C6 is larger; $d1_{C\text{-}B}$ is the distance of C3-C4 or C1-C6, and the distances between C2 and its neighboring C atoms are represented by $d2_{C\text{-}B}$.

Phase	Pressure (GPa)	$d_{B\text{-}H}$ (Å)	$d_{C\text{-}B}$ (Å)		$d_{C\text{-}C}$ (Å)		$d_{H\text{-}H}$ (Å)	$d_{C\text{-}H}$ (Å)
			$d1_{C\text{-}B}$	$d2_{C\text{-}B}$	$d1_{C\text{-}C}$	$d2_{C\text{-}C}$		
phase- α	0	1.208	1.626	1.626	1.529	1.541	2.10	1.097
	5	1.202	1.619	1.619	1.524	1.535	1.933	1.098
	10	1.193	1.612	1.612	1.513	1.527	1.833	1.094
phase- β	20	1.185	1.618	1.563	1.523	1.500	1.753	1.085
	50	1.169	1.552	1.552	1.479	1.475	1.614	1.072
	100	1.150	1.468	1.548	1.416	1.454	1.502	1.063
